# A Prospective Comparison of Insertion Characteristics of Laryngeal Mask Airway (LMA) ProSeal® Using Rotation Techniques vs Standard Techniques in Adults Undergoing Elective Surgery

**DOI:** 10.7759/cureus.37976

**Published:** 2023-04-22

**Authors:** Aadhar Khutell, Tanvi Grover, Apoorva Singh, Anita Seth, Mansha Madan, Kulbhushan Yadav

**Affiliations:** 1 Anesthesiology and Critical Care, Vardhman Mahavir Medical College and Safdarjung Hospital, New Delhi, IND; 2 Critical Care, Royal Preston Hospital, Preston, GBR

**Keywords:** 180 degree rotation, reverse rotation, 90 degree rotation, rotational technique, lma proseal, airway management

## Abstract

Background: Airway management is the most essential skill in Anesthesiology and the inability to secure the airway is one of the most common reasons for anesthesia-related morbidity and mortality. This study aimed to evaluate and compare the insertion characteristics of laryngeal mask airway (LMA) ProSeal insertion using the standard introducer technique, 90-degree rotation technique, and 180-degree rotation technique in adult patients undergoing elective surgery.

Material and method: This prospective, interventional, randomized, comparative study was carried out in the Department of Anesthesia and Intensive Care, Vardhman Mahavir Medical College & Safdarjung Hospital, New Delhi, after obtaining hospital ethical committee approval for 18 months of duration. Patients in the age group 18-65 years, of either gender, fulfilling American Society of Anesthesiologists grade I or II criteria, scheduled for elective surgery under general anesthesia with controlled ventilation using the LMA ProSeal were included in the study. The patients were randomized into three groups: Group I - Standard Introducer technique (n=40); Group NR - 90-degree rotation technique (n=40); Group RR - 180-degree rotation technique or back to front (airway) technique (n=40).

Result: In this study, the majority (73.3%) of the patients were females with 31 patients in group I, 29 patients in group NR, and 28 patients in group RR. A total of 26.67% of male patients were included in the study. No significant difference in the gender distribution of the three groups was seen in the study. There was no incidence of failure in ProSeal laryngeal mask airway (PLMA) insertion in the NR group, while it was 2.50% in group I and 7.50% in group RR but the difference was not statistically significant. A statistically significant difference was seen in the incidence of LMA ProSeal blood staining (p=0.013). In the post-anesthesia care unit at 1 hour, the incidence of sore throat was 10% in patients with the NR group, 30% in the I group, and 35.44% in the RR group which was statistically significant.

Conclusion: The study concluded that the 90-degree rotation technique was superior to both the 180-degree rotation and the introducer technique in adult patients in terms of insertion time, ease of insertion score, manipulation requirement, blood staining of PLMA, and post-op sore throat.

## Introduction

The laryngeal mask airway (LMA) device was first designed with the expectation of creating a device that diminished adverse anatomical and physiological events associated with upper airway manipulation. It has been shown to have beneficial effects over intubation by avoiding invasion of the larynx and trachea thereby reducing respiratory effects and cardiovascular stimulation [1]. Plasma concentrations of adrenaline and noradrenaline are higher following tracheal intubation than LMA insertion [2]. Once in situ, it occupies a relatively afferent nerve-deficient area around the glottis that is well adapted to dealing with foreign bodies such as boluses of food. This benefit extends into the postoperative period [3]. It has been suggested that postoperative analgesic requirements are reduced compared to intubation, probably due to related reduced central sensitization [4].

Using supraglottic airway devices comes with many advantages including speed and reliability of placement, the requirement of a lower skill set, removal of the need for additional equipment, more hemodynamic stability with insertion and removal along with a lower risk of airway trauma [5]. Patients with an LMA require a significantly less anesthetic agent to maintain the depth of anesthesia than those who are intubated [6].

Over 20 technique variations have been developed [1] to improve the insertion of the LMA ProSeal in patients; with an introducer, pharyngoscopic, 90-degree rotation technique, 180-degree rotation technique, suction catheter, gum elastic bougie, and fiberscope. The insertion eases with the conventional introducer technique since it occupies less space than the finger, directs the cuff around the oropharynx inlet, and facilitates full depth of insertion. However, it is reported to be more difficult due to impaction at the base of the mouth and folding of the cuff [7]. The incidence of trauma depicted by blood staining of LMA and sore throat can be significantly reduced by avoiding the contact of the device with the palate and pharyngeal wall with the rotation techniques [5].

In 2012, Kumar et al. stated that the 180-degree rotation method of LMA insertion was comparable to the standard technique and can be considered in adults when encountering difficulty and repetitive failures with standard LMA insertion techniques. The incidence of trauma in the rotational technique was remarkably lower at 6% compared to 28% with the standard LMA insertion technique [8].

The 90-degree rotation technique does not require an additional device or finger insertion in the oral cavity. Sore throat is not solely dependent on pharyngeal trauma but is multifactorial: use of lubrication, maintaining LMA cuff pressure, and user’s skill. A faster and improved insertion technique may be lifesaving in the emergency difficult airway scenario [8].

There is a paucity of literature comparing the insertion characteristics of rotational techniques with standard introducer techniques in adults. The primary objective of the study was the first attempt insertion success rate. The secondary objectives were insertion time, overall success rate, ease of insertion, number of attempts, hemodynamic response, oropharyngeal seal pressure, hemodynamic parameters, LMA ProSeal blood staining, and complications like sore throat, dysphagia, and hoarseness.

## Materials and methods

Study design

A prospective, comparative, interventional, and randomized study.

Sample size

It was conducted on 120 consenting adult patients of either gender after obtaining hospital ethical committee approval and registration with the clinical trials registry of India. The research was approved in a meeting of the Institute of Ethics Committee of Vardhman Mahavir Medical College and Safdarjung Hospital for all ethical purposes with serial number - 100.

Insertion of the ProSeal LMA is more successful with the 90-degree rotation technique as observed by Jeon et al. [9]. The study observed that the success rate at first insertion was greater for the 90-degree rotation technique group than for the standard technique group (100% vs 83%, respectively; P = 0.003). These values were taken as reference and a difference of 17% for the 180 rotation technique was assumed as compared to the standard technique. The minimum required sample size with 80% power of the study and a 5% level of significance was 39 patients in each study group. Hence the total sample size taken was 120.

Inclusion criteria

Patients in the age group of 18-65 years of American Society of Anesthesiologists (ASA) grade I and II scheduled for elective surgery of fewer than two hours duration under general anesthesia using LMA ProSeal for airway management.

Exclusion criteria

Patients with an anticipated difficult airway (Malampatti 3-4, mouth opening < 2.5 cms, restricted neck movements), body mass index >30 kg/square m, history of systemic and metabolic disorders, recent upper respiratory tract infection, sore throat, history of asthma, risk of regurgitation and aspiration, and pregnancy were excluded.

Block randomization

Block randomization was done with a sealed envelope system. In this, 15 randomly generated procedure allocations were prepared within sealed opaque envelopes which assigned I, NR, and RR in five envelopes each; where I represented the group receiving standard introducer technique, NR represented the group receiving 90-degree rotation and RR represented the group receiving 180-degree rotation (reverse rotation). Once a patient gave consent to enter a trial, an envelope was opened and the patient was then allocated to a particular group ensuring blinding.

Technique

After standard intravenous induction with routine doses of propofol, fentanyl, and vecuronium, an appropriately sized ProSeal LMA (LMA® ProSeal™ Airway, Teleflex Medical Europe Ltd., Ireland) was selected as per the weight of the patient and inserted three minutes after vecuronium administration according to the randomized selected technique by the anesthesiologist trained for at least three months. Another anesthesiologist noted the parameters. The cuff of ProSeal LMA was fully deflated and flattened. A water-based gel was applied to the posterior aspect and lateral surfaces of the LMA for lubrication.

The patient was supine with the head on a pillow placed in the sniffing position. The patient’s mouth was opened. Holding the LMA ProSeal, its entire cuff was placed into the patient’s mouth via the midline approach without finger insertion and then rotated 90 degrees counterclockwise around the tongue and then advanced until the resistance of the hypopharynx was felt. Then it was rotated back to the midline while being advanced further ahead. For the RR group, the ProSeal LMA was turned in a 180-degree back-to-front position with the concavity facing toward the hard palate (like an airway). It was then advanced into the base of the hypopharynx until resistance was felt. At this point, the LMA was rotated 180 degrees anti-clockwise and positioned in the pharynx. The requirement of manipulations for the insertion of LMA ProSeal was noted. The manipulations were noted as: in and out movement/jaw thrust/chin lift/neck extension or flexion. The number of attempts of LMA ProSeal insertion was also noted. An insertion attempt was defined as the placement of LMA ProSeal in the mouth. A failed attempt was defined as its removal from the mouth because of failed passage, malposition, or ineffective ventilation. If SpO2<95%, mask ventilation was done before the next attempt. A maximum of two attempts were allowed before a failure was recorded in which case standard airway protocol was followed.

After the LMA ProSeal was positioned in the pharynx, the cuff of LMA ProSeal was inflated with air up to the maximum volume recommended to 60cmH_2_O pressure using a cuff pressure monitor and maintained at this pressure. The LMA ProSeal was fixed and taped.

Parameters noted

Insertion time was noted as the time from picking up the LMA ProSeal till the establishment of an effective airway. Ease of insertion was scored using the three-zero score based on tactile resistance and the number of attempts [10]. Hemodynamic parameters including heart rate (HR), systolic blood pressure (SBP), diastolic blood pressure (DBP), and mean arterial pressure (MAP) were recorded. Electrocardiography (ECG), oxygen saturation (SpO2), and EtCO2 were monitored and noted one min before insertion of LMA ProSeal and at one, three, and five minutes after insertion. Peak airway pressure (Paw) was noted at one, three, and five minutes after insertion. Oropharyngeal seal pressure was determined by the manometric stability technique. After surgery LMA ProSeal was removed and assessed for any visible blood noted as no blood, trace amount, or significant amount. An anesthetist blind to the method of insertion recorded postoperative adverse events in the post-anesthesia care unit (PACU) in one hour and on the first postoperative morning: sore throat, hoarseness, and dysphasia.

Statistical analysis

Data were entered in Microsoft Excel 2010 (Microsoft Corp., Redmond, USA) and analysis was done by using the program Statistical Product and Service Solutions (SPSS) (IBM SPSS Statistics for Windows, Version 21.0, Armonk, NY).

Categorical variables are presented in number and percentage (%) and continuous variables are presented as mean ± SD and median. The normality of data was tested by the Kolmogorov-Smirnov test.

Quantitative variables were compared using analysis of variance (ANOVA) between the three groups while qualitative variables were compared using the Chi-square test.

## Results

The study population was comparable on demographic parameters, age, gender, and anthropometric parameters - height, weight, and BMI. A majority (73.3%) of patients were females. As the study was conducted in elective cases posted for surgery, the majority of patients were ASA grade 1. The majority (66.67%) of the patients in all the groups had ProSeal laryngeal mask airway (PLMA) size 4; with 65% of patients in group I, 72.5% of patients in group NR, and 62.5% of patients in group RR. Only 33.33% of total study subjects had PLMA size three. No significant difference in the PLMA size of the three groups was seen in the study (p>0.05).

All the baseline hemodynamic parameters were comparable between the three groups. A declining HR from baseline to five minutes after insertion in all groups except NR which showed a slight increase in HR at one minute and five minutes but it was not significant. A declining trend was seen in the mean value of SBP from baseline to five minutes after insertion in all the groups with a slight increase in value in group I at five minutes after insertion. Similarly, a declining trend was seen in the mean value of DBP from baseline to five minutes after insertion in all the groups with slight upward fluctuations at the end in all three groups which was not statistically significant. A similar trend was observed with MAP. A similar increasing trend was observed in the mean values of EtCO2 in all the groups with comparable airway pressures at one, three, and five mins post PLMA insertion.

Table 1 shows insertion time which was found significantly shorter in group NR as compared to group I (p=0.0001) and RR (p=0.0001). Insertion time was significantly longer in group RR as compared to I (p=0.0001) and NR (p=0.0001). The first attempt insertion success rate was comparable between the three groups with 87.50% in the NR group as compared to 85% in the RR group and 80% in group I. The number of attempts was comparable between the three groups. A second attempt was required in 17.50% of cases in group I, 12.50% of cases in group NR, and 7.50% of cases in the RR group.

**Table 1:** Insertion time I = standard introducer technique, NR = 90-degree rotation technique, RR = 180-degree rotation technique

Insertion time (secs)	Groups			Total	P value	I vs NR	I vs RR	NR vs RR	
	Standard I (n=39)	90 degree NR (n=40)	180 degree RR (n=37)						
Mean ± SD	26.87 ± 4.47	21.57 ± 2.87	29.23 ± 3.19	25.8 ± 4.79	<0.0001	<0.0001	0.009	<0.0001	
									Median (IQR)	26.46 (23.55-30.92)	20.88 (19.172-23.252)	29.16 (26.545-32.045)	26.08 (21.238-29.57)	

Table 2 shows group NR had significantly better ease of insertion scores than group I (p=0.017) and group RR. (p=0.0002) Also, a significantly smaller number of patients (15%) required manipulations in the NR group than in the I group (37.50%) (p=0.022) and the RR group (45%) (p=0.003). Out of 15, six, and 18 cases requiring manipulation in groups I, NR, and RR respectively, 100% of them required in and out movement. Chin lift was required in 66.67% of cases in group I, 0% in group NR, and 50% in group RR respectively. It was used in 48.72% of all cases requiring manipulations that were statistically significant (p<0.05). A significantly lower number of chin lift types of manipulation was required in group NR as compared to group I (p=0.012). Flexion or extension was required in 26.67% of group I, 0% in group NR, and 22.22% in group RR respectively. It was used in 20.51% of all cases requiring manipulations (p>0.05). Jaw thrust was required in 60% of all cases in group I, 0% in group NR, and 50% in group RR respectively. It was used in 46.15% of all cases requiring manipulations that were statistically significant (p<0.05). A significantly lower number of jaw thrust types of manipulation was required in group NR as compared to group I (p=0.019).

**Table 2:** Ease of insertion score I = standard introducer technique, NR = 90-degree rotation technique, RR = 180-degree rotation technique

Ease of insertion score	Groups			Total	P value	I vs NR	I vs RR	NR vs RR
	Standard I (n=40)	90 degree NR (n=40)	180 degree RR (n=40)					
0	1 (2.50%)	0 (0%)	3 (7.50%)	4 (3.33%)	0.002	0.017	0.307	0.0002
1	7 (17.50%)	5 (12.50%)	3 (7.50%)	15 (12.50%)				
2	13 (32.50%)	4 (10%)	18 (45%)	35 (29.17%)				
3	19 (47.50%)	31 (77.50%)	16 (40%)	66 (55%)				
Total	40 (100%)	40 (100%)	40 (100%)	120 (100%)				

The median oropharyngeal seal pressure in group I was 29 cm of H_2_O, in group NR was 31.50 cm of H_2_O, and in group RR was 31 cm of H_2_O as shown in Table 3. Oropharyngeal seal pressure was significantly higher in both rotational groups NR and RR as compared to group I (p<0.001) but was not clinically significant. But a comparison of oropharyngeal seal pressure between group NR and group RR was not statistically significant with a p-value>0.05.

**Table 3:** Oropharyngeal seal pressure I = standard introducer technique, NR = 90-degree rotation technique, RR = 180-degree rotation technique

Oropharyngeal seal pressure (cm H_2_O)	Groups			Total	P value	I vs NR	I vs RR	NR vs RR	
	Standard I (n=39)	90 degree NR (n=40)	180 degree RR (n=37)						
Mean ± SD	29.38 ± 2.86	31.5 ± 1.93	31.78 ± 2.46	30.88 ± 2.65	0.0004	0.001	0.0004	0.665	
									Median (IQR)	29 (27-32)	31.5 (30-33)	31 (29.5-34)	31 (29-33)	

A significant difference was noted in the incidence of PLMA blood staining (p=0.013) as shown in Table 4. PLMA was found blood-stained in 27.50% of patients in group I and 32.50% of patients in group RR whereas only 7.50% of patients in group NR had blood staining which was statistically significant. No case had significant blood staining while 22.50% of the total cases had trace blood staining of PLMA. Trauma to lips, teeth, gums, or oral cavity was not observed in any of the patients in any of the groups in this study.

**Table 4:** PLMA blood staining I = standard introducer technique, NR = 90-degree rotation technique, RR = 180-degree rotation technique, PLMA = ProSeal laryngeal mask airway

Blood staining	Groups			Total	P value	I vs NR	I vs RR	NR vs RR	
	Standard I (n=40)	90 degree NR (n=40)	180 degree RR (n=40)						
Nil	29 (72.50%)	37 (92.50%)	27 (67.50%)	93 (77.50%)	0.013	0.037	0.626	0.01	
									Traces	11 (27.50%)	3 (7.50%)	13 (32.50%)	27 (22.50%)	
Significant	0 (0%)	0 (0%)	0 (0%)	0 (0%)					
Total	40 (100%)	40 (100%)	40 (100%)	120 (100%)					

Group NR had significantly less incidence of sore throat 1 hour after surgery than group I and RR (p<0.05) as shown in Table 5. All the patients reporting sore throat stated it to be mild. No significant difference was observed in the incidence of the sore throat between the three groups on the first post-op morning. No other adverse events were noted in any of the groups.

**Table 5:** Incidence of sore throat I = standard introducer technique, NR = 90-degree rotation technique, RR = 180-degree rotation technique, PACU: post-anesthesia care unit

Adverse events		Groups			Total	P value	I vs NR	I vs RR	NR vs RR	
		Standard I (n=39)	90 degree NR (n=40)	180 degree RR (n=37)						
Sore throat in PACU (1 hour)	None	27 (69.23%)	36 (90%)	24 (64.86%)	87 (75%)	0.023	0.027	0.686	0.012	
										Present	12 (30.77%)	4 (10%)	13 (35.14%)	29 (25%)	
	Sore throat (first post-op morning)	None	33 (84.62%)	37 (92.50%)	33 (89.19%)					103 (88.79%)	0.537	0.311	0.737	0.705	
Present		6 (15.38%)	3 (7.50%)	4 (10.81%)	13 (11.21%)									

Only three cases out of 117 experienced dysphagia in PACU (2.56% in group I, 0% in group NR, and 5.41% in group RR). No significant difference was observed in the incidence of dysphagia between the three groups in the post-op care unit and at first post-op monitoring. In the PACU and at first post-op monitoring, none of the patients had hoarseness.

## Discussion

In this prospective, comparative, interventional, and randomized study conducted in 120 adult patients, we compared the insertion characteristics of LMA ProSeal insertion using the standard introducer technique (group I), 90-degree rotation technique (group NR), and 180-degree rotation technique (group RR) we found similar first attempt success rate between the three groups. Our study did not show a statistically significant difference between 90-degree and 180-degree rotation techniques over the standard introducer technique in the first attempt insertion success rate in line with trials conducted by Park et al. and Haghishi et al. [11,12]. Both compared 180-degree rotation in adult patients with standard technique and found no meaningful advantage of 180-degree rotation. However, studies done by Dhulked et al. [13], Jeon et al. [9], and Hwang et al. [5] noted significantly better first-attempt success rates with 90-degree rotation compared to the standard technique.

The number of attempts was also comparable between the three groups. Overall successful insertion rate was 96.67% with no failure in the 90-degree rotation group, one failure in the standard introducer group, and three failures in the 180-degree rotation group. There was no incidence of failure in PLMA insertion in the NR group, while it was 2.50% in group I and 7.50% in group RR but the difference was not statistically significant.

There was no significant difference in overall successful insertion rates. The single failure with the standard introducer was due to repeated curling of the PLMA which could be attributed to impaction at the back of the mouth. Two of the three failures in 180-degree rotation were due to the inability to rotate the PLMA size 4 inside the oral cavity. The third patient had a large tongue which may have obscured the cuff rotation. These patients were intubated as per protocol and were not evaluated for post-operative adverse events.

The mean insertion time in the 90-degree rotation group was 21.57 ± 2.87 seconds, in the standard introducer group was 26.87 ± 4.47 seconds, and in the 180-degree rotation group was 29.23 ± 3.19 seconds. Insertion time was significantly shorter in the group with the 90-degree rotation technique as compared to the standard introducer group and 180-degree rotation group (p <0.05) as seen by Jeon et al. [9] comparing the 90-degree rotation technique with the standard technique. However, studies were done by Haghishi et al. [12] and Kuvaki et al. [14] on classic and softseal LMA found insertion time to be significantly less with 180-degree rotation over the standard technique. This was likely due to PLMA being bulkier making it difficult to completely rotate it by 180 degrees in the mouth.

Ease of insertion was assessed using ease of insertion scoring based on tactile resistance instead of insertion time as in studies by Jeon et al. [9], Yun et al. [15], and Dhukhed et al. [13] and our study showed that the 90-degree rotation group had a significantly better score than both 180-degree rotation technique and standard introducer technique using this scoring system.

A statistically significant difference was seen in the manipulation requirement. A significantly less number of patients (15%) required manipulations with the NR group as compared to the I group (37.50%) and group RR (45%). The manipulation requirement for PLMA insertion was statistically significant between the NR group and I group as well as the NR group and RR group.

Oropharyngeal seal pressure was significantly higher in groups with 90-degree rotation and 180-degree rotation as compared to the standard introducer group indicating that both 90-degree and 180-degree rotational techniques provide a better seating of the LMA ProSeal than the introducer group. Jeon [9] found no significant difference between the standard (25 ± 7 mmHg) in standard and 90-degree rotation (27 ± 6 mmHg) but inflated the LMA cuff to 50% of the recommended maximum volume.

A statistically significant difference was seen in the incidence of LMA ProSeal blood staining (p=0.013). A significantly less number of cases (7.50%) had trace blood-stained PLMA with a 90-degree rotation technique than the standard technique (27.50%) and 180-degree rotation technique (32.50%). None of the cases had significant blood staining of the PLMA. Multiple studies [5,9,13] on PLMA have compared 90-degree rotation with standard and arrived at a similar conclusion. Haghishi et al. [12] conducted a study on classic LMA and found a significantly lower incidence of blood staining (18%) with 180-degree rotation than the standard technique. We could not reproduce similar results as PLMA, despite having a smooth surface, is bulkier than classic LMA and its rotation would cause more injury due to friction on a complete rotation. This problem is resolved with 90-degree rotation as it involves lesser movement (thereby lesser friction) of PLMA inside the oral cavity.

The group 90-degree rotation had significantly less incidence of mild sore throat 1 hour after surgery than the standard introducer group and 180-degree rotation group. On the first postoperative morning, none of the patients had a sore throat. Studies by Hwang et al. [5], Jeon et al. [9], and Dhulkhed et al. [13] compared 90-degree rotation with standard technique and found similar results (8% vs 25%, 15% vs 38.4%, and 12% vs 33% respectively). The etiology of postoperative sore throat is unclear but has been associated with the female sex, insertion technique, use of lubricants, and lack of humidification of airway gases [16]. The pathophysiology seems to be neurogenic inflammation [17].

Strength

Randomization, single anesthesiologist preventing inter-observer bias, and a dearth of studies on PLMA with 180 in adults.

Limitations

Our study has some limitations. The study was conducted in adult patients of ASA I and ASA II‎ having normal airways for elective surgery, hence our findings may not apply to patients with a difficult airway. It was not possible to blind the technique of insertion from the anesthesiologist doing the observations causing a source of potential bias. The PLMA was inserted in anesthetized paralyzed patients and so the results may vary in spontaneously breathing patients. The type of manipulation was based on the Anesthesiologist’s assessment. Airway patency was not assessed with a fiberoptic bronchoscope.

## Conclusions

The first attempt insertion success rate was found comparable between the standard and rotational techniques. The 90-degree rotation technique was superior to both 180-degree rotation and the introducer technique in adult patients in terms of insertion time, ease of insertion score, manipulation requirement, blood staining of PLMA, and post-op sore throat. Since the 90-degree rotation group had all the best results in insertion characteristics, and the least complications, it may be considered the preferred technique for LMA ProSeal insertion in adult patients with normal airways.
